# The Effectiveness of Oral Rehydration Solution at Various Concentrations as a Storage Media for Avulsed Teeth

**Published:** 2013-01-20

**Authors:** Tahereh Eskandarian, Samaneh Badakhsh, Tahereh Esmaeilpour

**Affiliations:** 1 Department of Pediatric Dentistry, Dental School, Shiraz University of Medical Sciences, Shiraz, Iran; 2 Department of Pediatric Dentistry, Dental School, Zanjan University of Medical sciences, Zanjan, Iran; 3 Laboratory of Stem Cell Research, Department of Anatomy, Shiraz University of Medical Sciences, Shiraz, Iran

**Keywords:** Tooth Avulsion, Culture Media, Rehydration Solutions, Periodontal Ligament

## Abstract

**Introduction:**

Following avulsion, the periodontal ligament (PDL) cells are at risk of necrosis. To achieve a favorable survival prognosis, the PDL cells must be kept viability. Therefore, immediate replantation is considered as the treatment of choice and in case it is not possible, storing the tooth in an appropriate storage media should be considered. Oral Rehydration Solution (ORS) is a glucose-electrolyte solution which can keep the optimal osmolality as well as pH and can even provide nutrients which are necessary for cellular growth. The present study aimed to evaluate the effectiveness of different concentrations of ORS in maintaining the viability of the PDL cells at different time points.

**Materials and Methods:**

PDL cells were obtained from healthy extracted human premolars. Then, 8×10³ cells were seeded in each well of 96-well plate. Afterwards, each well was treated with ORS in three different concentrations and DMEM for 1, 3, 6, and 9 hours. Cell viability was determined by using the MTT assay. One way-ANOVA and post hoc (LSD) test were used for comparing the study groups.

**Results:**

According to the results, 25% and 50% concentrations of ORS were more effective in preserving the PDL cell viability and could maintain 79.98% and 68.34% of the PDL cells, respectively, at least for the last experimental time point (up to 9 hours).

**Conclusions:**

Therefore, our findings indicate that ORS might be a suitable storage medium for avulsed teeth.

## 1. Introduction

Tooth avulsion is a complex traumatic injury characterized by severe damage to the periodontal and pulpal tissues. It most frequently occurs in boys, moreover, 8-12 years old children are more affected. This is probably due to low mineralized alveolar bone and loose periodontal ligament, [1, 2]. According to literature, the prevalence of avulsion is 1-16% of traumatic injuries in the permanent dentition [3, 4]. Immediate replantation is considered as the ideal treatment for this type of trauma, but it is not always possible. Therefore, keeping the tooth in an appropriate transporting media is needed [1, 3, 4]. It has been shown that storing the tooth in a proper media is more important than the extra alveolar period [5, 6, 7]. Up to now, several known storage media, such as saliva (buccal vestibule), saline, milk, culture media, Hank’s Balanced Salt Solution (HBSS) and Viaspan, have been examined and some media have been tested recently (i.e. coconut water, propolis, egg albumen, Gatorade, green tea) [4, 5, 7]. Soder et al. reported that the dry environment is detrimental for PDL cells and after 2 hours in such conditions, no other viable cells are present [2]. Many studies have demonstrated the efficacy of culture media in preserving cell viability [8, 9]. Ashkenazi et al. revealed that after 24 hours at room temperature, Viaspan and culture media were the most effective media in maintaining PDL cells viability [10]. Andreasen showed that after 5-7 days storing in culture media, the amount of inflammatory resorption had remarkably decreased [11].

Oral Rehydration Solution (ORS) is a simple, inexpensive glucose and electrolyte solution which has been widely used in treatment of dehydration resulting from diarrhea of any etiology and is considered appropriate for all ages. The ORS formula contains sodium chloride, potassium chloride, glucose, and trisodium citrate [12]. The present study aimed to determine the effectiveness of different concentrations of ORS, used as a storage media in preserving PDL cells viability.

## 2. Materials and Methods

PDL cells were obtained from premolars extracted for orthodontic purposes which were clinically healthy and had healthy gingiva (i.e. not inflamed). The teeth were rinsed with normal saline 3 times. Then, they were transferred to the laboratory within 30 minutes. The transporting media consisted of Dulbecco’s Modified Eagle Medium (DMEM), 10% Fetal Bovine Serum (FBS), 1% antimycotic, and 1% gentamycine. Under laminar flow hood, the crown of the teeth was took with sterile forceps and again rinsed with Phosphate Buffer Saline (PBS) for 3 times. Then, the periodontal tissue was scrapped with a sterile scalpel blade no.11 from one third of the middle root surface. After splitting the tissues to small pieces, they centrifuged for 5 minutes at 1200 rpm and were treated with 1mL colagenase type I ( 4 mg/mL) as well as 1 mL dispase (3 mg/mL); they were then incubated at 37ºC (95% air and 5% CO2) for 1 hour. The cellular suspension was cultured in culture media containing DMEM, 1% L-glutamin, 10% FBS, 1% antimycotic, and 1.8% Human AB Serum. Finally, passage 3-4 was used.

By using still water, ORS diluted to three different concentrations (25, 50 and 100%). Other media which were evaluated in this study were DMEM, as positive control, and the negative controls was a media free.

In the present study, 8×10³ cells were seeded in each well of the 96-well plate. Each experimental storage media were repeated for 6 times. Different incubation periods were 1, 3, 6, and 9 hours. The plates were incubated overnight. After that, the culture media was replaced with 100 µL of the 5 different groups for the experimental time intervals (1, 3, 6, 9 hours).

The viability of the cells was determined by MTT assay; that is, 150 µL of MTT (20µL/mL) was added and cultures were then incubated for 4 hours and were replaced with 150 µL of DMSO afterwards. Optical density was measured at 492 nm with ELISA plate reader.

One way-ANOVA and post hoc (LSD) test were used for comparison between groups. Moreover, P-value <0.05 was considered as statistically significant.

## 3. Results

**Figure 1.**
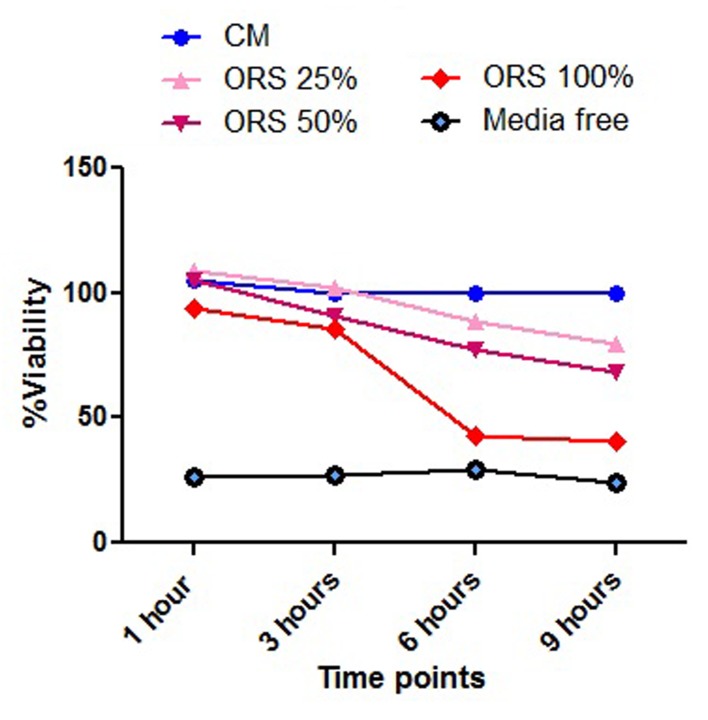
Graph shows the PDL cell viability.

As shown in Table 1, after 1hr, DMEM and ORS in 25% (P=0.381) and 50% (P=0.944) concentrations were similar and were significantly better than the other subgroups i.e., the best experimental media. Among different concentrations, ORS in 25% and 50% concentration were more effective, at least up to 9 hours. Though 100% concentration of ORS also had the ability of maintaining the cell viability, it had the lowest capacity when compared to the different concentrations. Graph shows the viability of the PDL cells (Figure 1).

**Table 1.** Percentage of viable PDL cells [Mean(SD)] in different storage media at several time points

Storage Media	Mean ± SD (1 h)	Mean ± SD (3 h)	Mean ± SD (6 h)	Mean ± SD (9 h)
**DMEM a**	105±3.07	100±2.09	100±2.52	100±6.23
**ORS 25% b**	109±2.78	102±3.14	88.84±1.62	79.98±8.67
**ORS 50%**	105±3.56	90.76±3.13	77.09±2.89	68.34±3.61
**ORS 100%**	94.2±2.74	85.76±3.43	42.84±3.82	40.39±0.93
**Negative control c**	26.72±0.54	27.38±0.49	29.03±1.07	24.49±2.84

^a^DMEM: Dulbecco’s Modified eagle Medium,

^b^ORS: Oral Rehydration Solution,

^c^Negative Control: Media free condition.

## 4. Discussion

Treatment of an avulsed tooth is a great challenge that dentists deal with. The most crucial factor which influences the final prognosis is existence of viable PDL cells. Therefore, immediate replantation is considered as the treatment of choice. If this is not possible, the tooth should be kept in a proper storage media [5, 7, 12]. The storage media should be able to preserve the cell viability and also be readily available [13]. Many studies have demonstrated that culture media (i.e. DMEM) is highly potent for preserving the PDL cells [7, 12]. It was reported that this culture media is effective at least for 48 hours [14]. Our findings demonstrated that in all experimental time points, DMEM could result in 100% viability.

According to other studies, moist environment is much better than dry media [15]. Although the negative control group in our study was a media free condition, there was enough humidity in incubator to keep the cells’ viability in all experimental times.

The other storage media tested in this study was ORS; this is a glucose-electrolyte solution whose compositions keep the optimal osmolality as well as pH and can even provide nutrients. It has been observed that pH and osmolality of the storage media are important for cell growth and should be in the pH range of 6.6-7.8 and the osmolality range of 230-400 mOsm [16]. The osmolality and pH of ORS is 270 mOsm/L and 7.8, respectively which makes it suitable for cellular growth. In addition, ORS is easily available and inexpensive.

In the present study, ORS was evaluated in 3 different concentrations. Mousavi et al. observed that by using ORS, the viability of the PDL cells was maintained for at least 12 hours and was similar to HBSS [13]. Our results indicated that although different concentrations of ORS have the possibility of preserving PDL cell viability, the 25% and 50% concentrations are more effective and could on average preserve 79.98% and 68.34% of the PDL cells, respectively. As shown in Table 1, within one and three hours after treating the cells with ORS, the percentage of the viable cells were over 100% which demonstrates that some cellular proliferation has occurred. According to these findings, we can claim that ORS not only preserves cell viability, but also it has regenerative ability. The 100% concentration of ORS was considered as the worst concentration, which might be because of hyperosmotic situation.

## 5. Conclusion

The obtained results show that ORS can be an alternative media for storing the avulsed teeth. Naturally, further in vivo studies are needed for absolute confirmation.
